# Construction of life-and-death education contents for the elderly: a Delphi study

**DOI:** 10.1186/s12889-022-13197-7

**Published:** 2022-04-21

**Authors:** Lei Lei, Ya Lu, Hongyan Zhao, Jing Tan, Yu Luo

**Affiliations:** 1 School of Nursing, Army Medical University (Third Military Medical University), No. 30 Gaotanyan Street, Shapingba District, Chongqing, 400038 P. R. China; 2 Xiaolongkan Community Health Service Center, No.4 Xiaolongkan Street, Shapingba District, Chongqing, 400030 P. R. China

**Keywords:** Life-and-death, Construction, Palliative-and-hospice care, Aged, Delphi

## Abstract

**Background:**

Life-and-death education is intimately related to palliative-and-hospice care. It should be implemented among groups of all ages, especially for the elderly. This study aims to establish expert consensus on a set of scientific and systematic life-and-death education contents for the elderly and provide reference for the practice on the elderly.

**Methods:**

This study designed three rounds of expert consultation by using a Delphi method. A panel of 22 experts from the fields of palliative-and-hospice care, life-and-death education, geriatric nursing, humanities and ethics, and geriatric psychology participated in the study.

**Results:**

This study finally reached expert consensus on the contents of life-and-death education for the elderly, containing 4 first-level items, Life-and-death literacy promotion in the elderly; Life-and-death concept establishment of the elderly; Life-and-death planning of the elderly; Life-and-death thoughts of the elderly with affiliated 9 second-level items, and corresponding 23 detailed third-level items.

**Conclusions:**

The life-and-death education contents for the elderly offer a basis for publicity for health professionals, promote dialogues on death, preparation, and planning for death and dying. The life-and-death education contents system was clear in coherence containing definite and comprehensive contents, which enriched life-and-death education resources globally. The results could assist in the planning of palliative-and-hospice care services to improve quality of death of the elderly.

## Background

The population and proportion of the elderly have increased rapidly in recent years, and virtually all countries are experiencing population ageing [1]. According to the World Health Organization (WHO), the number of the global population aged over 60 and above is estimated to be doubled to two billion in 2050, and 80% of the elderly are in low and middle-income countries [2]. China is now one of the fastest aging countries [3]. The results of China's seventh census indicate that the population aged above 60 is approximately 264 million people [4].

With the expansion of people’s life span, the quality of life and death are of vital significance [3]. Medicine is both scientific and humanistic [5] because it not only cures diseases and prolongs life, but also brings about dignity and rights of decision to the elderly. To improve the quality of life of the elderly worldwide, WHO has proposed programs of healthy ageing, active ageing, and others [3]. Palliative-and-hospice care has become one of the principal measures to ease the pressure on medical care and to improve quality of life of the older people [6]. Palliative-and-hospice care provides physical, psychological, social, and spiritual care as well as humanistic care for end-of-life or elderly patients before their deaths, so that their pains and discomfort symptoms can be alleviated, by improving the quality of life and helping patients relax and leave with dignity [7, 8]. With the promotion and application of this mode of care, the topic of death is inevitable for the elderly. On the other hand, as the age increases, the body experiences weakness in both physical strength and decline in physical abilities [9]. Moreover, experience of the loss of important persons occurs more frequently [10]. The factors turn death into an inescapable fact for the elderly. Additionally, the threats of chronic diseases and infectious diseases, such as cancer, COVID-19, and Ebola keep reminding us that death is close at hand [11]. If older people know less about death, they are more likely to experience aggravated suffering about death, which is not conducive to the physical and mental health of them [12].

Unfortunately, the topic of death is often avoided or denied in eastern and western cultures [13]. Human attitudes and behaviors are deeply rooted in culture [14]. Actually, most of people are usually negative to the topic of death. They are afraid of mentioning it and reluctant to discuss dying matters [15]. It is therefore that people focus more on life planning, healthy birth, excellent education, and a better life whereas death is rarely mentioned. China ranks 9th from the bottom in the 2015 Quality of Death Index Ranking released by The Economist Intelligence Unit [16], which also reveals that Chinese people are far from the goal of good quality of death. Regarding the issue of death, Western society has carried out research related to thanatology as early as the 1920s [17]. Death awareness campaigns arose in the United States in the 1960s [18]. Courses of life-and-death education were instructed in colleges and universities in the 1980s [19]. As Herman Feifel stated in the early days of the death awareness movement that the attitude towards death affects the content and quality of our lives [20]. The quality of death in Western countries is generally better than that in Mainland China. Therefore, how to construct a set of life-and-death education content system integrated with culture, allowing the elderly to face death squarely and build a scientific concept on death has become the key to improving the quality of life and death in contemporary society and medical background.

Life-and-death education offers death information which is helpful for the educated to understand the finiteness of life and the naturalness of death objectively, so as to cherish life and appreciate life [21]. Life-and-death education transmit not only knowledge and skills related to death, broaden the understanding of dying, death, and bereavement, it is also thought provoking. "Acceptance the naturalness of life" and "development of the concept of death" contribute to a better self-realization [10, 22]. In addition, life-and-death education can also provide more options for dying and health care.

Life-and-death education highlights the review of the meaning of life and the discussion of death and helps to better promote advance directives or establish a advance care planning, allowing to achieve the goal of palliative-and-hospice care and improve the quality of death [23, 24], which is intimately related to palliative-and-hospice care. Based on the terror management theory, life-and-death education can activate reminders of death or mortality salience effect, trigger a distal defense mechanism to improve the meaning of life [25, 26]. As the concept of death influenced by many factors [27, 28], especially culture, the development of life-and-death education in accordance with local conditions can benefit the elderly to recognize relevant knowledge and change their negative attitudes towards death escapes or denies. Gradually, they recognize the finiteness of life, and then cherish the moment of life, live more valuable lifestyles, and select optimal medical treatments, thereby living an optimistic life and truly realizing active aging [18].

Life-and-death education should be implemented among groups of all ages, and it is particularly significant for the elderly [14]. However, the elderly has always been one of the groups that is easy to ignore in the research of life-and-death education [14]. Previous life-and-death education researches mostly focused on adolescents [29, 30], medical and healthcare professionals [31], and cancer patients [21] to explore the benefits of life-and-death education in reducing negative death emotions, managing the risk of commit suicide, and alleviating alexithymia [32, 33]. Most the education contents only focus on the sharing of living wishes and discussion of death recognition, while lacks contents of bereavement, mourning, and death preparations [34], and scientific construction of the life-and-death education contents, especially for the elderly. Therefore, this research attempts to establish expert consensus on a set of scientific and systematic life-and-death education contents for the elderly by Delphi technique. This project may provide a foundation for better implementation and promotion of life-and-death education among the elderly, and help improve the quality of death and well-being of the elderly.

## Methods

This study was approved by the Ethics Committee of the University (NO. 2021.06–03) on June 21, 2021. Delphi method has been widely used in education, health related fields which make full use of the wisdom of experts to construct and improve the new research content. This method is suitable for the purpose of this study. Delphi is a research method that achieves expert consensus through the anonymous and iterative multistage process [35, 36]. The trustworthiness of Delphi can be confirmed by credibility, dependability, confirmability, and transferability [37]. The credibility of Delphi can be gained by ongoing iteration and feedback to experts, which can be viewed as member checks [38]. Recruiting authoritative experts with in-depth research and interest in the topic will help increase the content validity and dependability of Delphi method [39]. Confirmability can be assessed through experts’ opinions collection, data analysis and detailed description of the consultation process [37]. Transferability of Delphi can be verified by application of the research findings [40].

### Expert selection

This study included experts in the fields of palliative-and-hospice care, life-and-death education, geriatric nursing, humanities and ethics, and geriatric psychology in the CNKI database, with the following inclusion and exclusion criteria: Inclusion criteria: 1. bachelor’s degree or above; 2. professional title qualification of intermediate or above; 3. work experience over 5 years; Exclusion criteria: 1. experts could not participate in this research due to personal reasons; 2. experts whose contact information were not available; 3. experts who had no practical experience of working with the elderly. Experts who meet the inclusion criteria, have conducted in-depth research and published articles in related fields. So the e-mail addresses and some phone numbers could be obtained from the database. Then the experts were invited to participate in the survey by e-mail, phone calls or text message, and they got the information about the content, purpose, and significance of the research in detail. A total of 23 experts agreed to participate in this Delphi study and follow-up questionnaires were sent to them and then returned after fulfillment by emails.

### Questionnaire design

The questionnaire for experts mainly consists of three parts: 1. personal sociodemographic information of the experts name, age, professional field, working years, and so on; 2. self-assessment of the familiarity to the questionnaire contents, including the overall familiarity. 3. expert evaluation form for the life-and-death education contents for the elderly. This part was the main body of assessment in the expert questionnaire. Based on retrieval of literature [41–43], books [44, 45], and websites of hospice [46–48] for the elderly, we found that education for the elderly should include physical, psychological, social, spiritual and end-of-life aspects at least. According to the purpose of this study and the previous research, a preliminary draft of the framework of life-and-death education contents for the elderly was made, and the importance of each part was assessed by experts with scores. A 5-point Likert scale method was employed to measure importance from 5 points to 1 point as "very important"-"very unimportant", in descending order of importance.

### Questionnaire procedures

The present study involved three rounds of questionnaires to the expert panel from July to November 2021. Experts did not communicate directly with each other. The researchers sorted out the opinions, suggestions and feedback of each expert and presented them anonymously on the next round of expert correspondence questionnaire. So the experts could get to each other. The first administration of questionnaires to the expert panel was completed in August. This was conducted in the form of emails, and researchers contacted the experts personally, collected their opinions and revised feedback. The first round of survey contained relatively open questionnaire items to seek expert opinions on the life-and-death education contents for the elderly. Seventeen educational contents were initially constructed. In addition to the scores evaluation, experts could also put forward their own professional opinions and suggestions for modification as well as the addition of other relevant contents to enrich life-and-death education system for the elderly.

According to the results of the first round of inquiries to the expert panel, life-and-death education contents for the elderly were revised and improved following relevant literature retrieval and discussions with professors and research team members. An expert evaluation form for the life-and-death education contents for the elderly was designed, including 4 first-level items, 10 second-level items, and 27 third-level items, and a second round of questionnaire to the expert panel was conducted, and the opinions and feedback from the experts were collected and analyzed.

Based on the opinions of the second-round administration and research purposes, the content system of life-and-death education for the elderly was modified with some deletion. As a result, an expert evaluation form of a third-round survey was generated containing 4 first-level items, 9 second-level items, and 23 third-level items, and the opinions and feedback from the experts were collected and analyzed.

### Data analysis

The returned data were analyzed using Excel and SPSS 24.0. Experts’ concern for this study was reflected in the positive coefficient of the expert panel, i.e., the rate of return of a questionnaire [21]. It is generally believed that the positive coefficient above 85% indicates good feedback of the survey from the expert panel. The representativeness of the experts in this research were presented as expert authority coefficient [21]. Generally, an authority coefficient over 0.70 indicates that the expert opinions are reliable and the experts are authoritative. The inclusion criteria for items in this study were the average importance score of each item evaluated by the expert panel > 4.00, the coefficient of variation < 0.20, and the approval rate > 80% [49]. Furthermore, based on some written opinions of the experts with literature retrieval, the proposed contents were discussed among the research team and improved by means of deletion, modification, supplementation, and merger.

## Results

### Expert sociodemographic information

The present research enrolled a panel of 23 experts at first from five fields of life-and-death education, palliative-and-hospice care, geriatric nursing, geriatric psychology, and humanities and ethics. The working years of experts are between 5 to 40 years, and more than 78% of experts have worked in the professional fields for more than 20 years. The sociodemographic details of the experts were presented as Table 1. And all the experts have experience working with the elderly.

**Table 1:** The socio-demographic information of the experts

Groups	Classification	Number of first-round	Percent (%)	Number of second-round	Percent (%)	Number of third-round	Percent (%)
Gender	Male	2	8.70	2	9.09	2	9.09
	Female	21	91.30	20	90.91	20	90.91
Age	30–39 years	3	13.04	3	13.64	3	13.64
	40–49 years	7	30.43	7	31.82	7	31.82
	50 years and over	13	52.17	12	54.54	12	54.54
Years of working	5 ~ 9 years	3	13.04	3	13.64	3	13.64
	10–19 years	2	8.70	2	9.09	2	9.09
	20–29 years	8	34.78	8	36.36	8	36.36
	30 years and over	10	43.48	9	40.91	9	40.91
Highest education	Master	5	21.74	5	22.73	5	22.73
	Doctor	18	78.26	17	77.27	17	77.27
Professional positions	Intermediate certificate	1	4.35	1	4.55	1	4.55
	Senior position	22	95.65	21	95.45	21	95.45
Supervisor	Doctor supervisor	13	56.52	12	54.55	12	54.55
	Master supervisor	8	34.78	8	36.36	8	36.36
	No	2	8.70	2	9.09	2	9.09
Specialist areas	Palliative-and-hospice care	6	26.09	6	27.27	6	27.27
	Life-and-death education	3	13.04	3	13.64	3	13.64
	Geriatric nursing	5	21.74	4	18.18	4	18.18
	Humanities and ethics	4	17.39	4	18.18	4	18.18
	Geriatric psychology	5	21.74	5	22.73	5	22.73

The present study involved three rounds of questionnaires to the expert panel. In the first round of Delphi, 23 experts returned questionnaires in time. In the second round, one expert failed to return the questionnaire due to her busy work, so she had to withdraw from the study, and the assessments from the 22 experts were collected. Also, all 22 experts gave feedback in the third round. In general, the response rate of the experts is more than 95% (> 85%), and the authority coefficient is more than 0.85 (> 0.70), indicating the authority of the experts and the credibility of the Delphi results. Experts were interested in the study through their comments. The main purpose of each round of questionnaires, the number of questionnaire items to be evaluated by experts, item revisions and expert opinions were presented in Table 2.

**Table 2:** The process of items revision

Round	Response rate %	Authority coefficient	Purpose	Total number of items	Number of qualified items	Number of modified items	Number of deleted items	Number of added items	Number of experts’ suggestions
The first-round	100.000	0.883	Explore	17	9	6	4	30	58
The second-round	95.650	0.898	Clarification	41	37	3	5	0	27
The third-round	100.000	0.896	Confirm	36	36	1	0	0	5

### Evaluation of life-and-death education contents for the elderly

In the first round of questionnaires to the expert panel, the items that did not meet the standards were deleted or modified, seen Table 3 for details. In addition to suggestions for changes in expression, items with overlapping contents were deleted. For example, the "various religions" contains the "Chinese traditional culture". Considering the cognitive acceptance of the elderly and the relevance of education contents for the elderly, the "Life-and-death concepts in traditional Chinese culture" (Confucianism, Taoism, and Buddhism)” were retained ultimately. "Organ transplantation and donation" was also deleted. In addition to the low approval rate by experts, organs of the elderly age naturally, and the age of organ donation generally does not exceed 65 years [50]. It is therefore that this content was not retained. The "Depression and suicide" of the elderly have not yet become the focus of experts’ attention, which differs from the western social background [51]. Based on the suggestions of the experts, the contents of life-and-death education was supplemented considering the characteristics of the elderly, and the second round of questionnaires was developed.

**Table 3:** Life-and-death education contents after expert consultation. (First-round)

Items	Mean	SD	CV	Approval rate %	Notes
1. Concepts of life and death (physical, psychological, social)	4.696	0.559	0.119	95.652	
2. Review of the meaning of life	4.783	0.518	0.108	95.652	
3. The psychological process and coping of death	4.783	0.518	0.108	95.652	
4. Life-and-death concepts in different religions	4.174	0.834	0.200	82.609	Delete (duplicate with 5.)
5. Life-and-death concepts in Chinese traditional culture	4.783	0.422	0.088	100.000	
6. Ethical and legal issues in euthanasia	4.435	0.945	0.213	91.304	Modify
7. Organ transplantation and donation	4.130	0.869	0.210	69.565	Delete
8. Depression and Suicide	3.783	1.242	0.328	73.913	Delete
9. Introduction to the Will	4.826	0.491	0.102	95.652	
10. Introduction to palliative-and-hospice care	4.913	0.288	0.059	100.000	
11. Introduction of living wills	4.696	0.876	0.186	95.652	
12. Funeral rites in various culture and religions	4.130	0.869	0.210	69.565	Modify
13. New Era Funeral Culture Initiative	4.348	0.832	0.191	86.957	Delete
14. Learn to communicate and express, life and death in peace	4.652	0.885	0.190	95.652	Modify
15. The emotional treatment of bereaved family members	4.609	0.941	0.204	91.304	Modify
16. Cultivate hobbies	4.522	0.994	0.220	86.957	Modify
17. Increase social participation	4.478	0.994	0.222	86.957	Modify

*Note*: *SD* Standard Deviation, *CV* Coefficient of variation

In the second round of questionnaires to the expert panel, the answers, modification and explanation of the first-round comments and a total of 41 items at three levels which needed to be evaluated by experts were presented. Then experts put forward some suggestions for revision. For example, "Advance Directives and Wills", both of which in Chinese have the meaning of end-of-life instructions and are likely to be confused by most elderly people who are first exposed to them. But advance directives and wills have different emphases, and it is not necessary to explain at the same time. So, according to experts' advice, wills and advance directives are split into two parts of “Planning before death or dying”. The content related to "Euthanasia" ultimately was not included in the education system. As euthanasia remains to be ethically controversial, and it is not permitted by law [52], experts considered that this part of content should be avoided in education for the elderly. "Funeral Ceremony" may originally derive from culture, customs, or religions, and it was integrated into "Chinese funeral Customs" in this study from the perspective of being easy to accept and practical for Chinese elderly. In addition to the deletion of items that did not meet the criteria, duplicate items were removed from the contents as per the proposal of the experts, and partial items were modified as well, as presented in Table 4.

**Table 4:** Life-and-death education contents after expert consultation. (Second-round)

Items of first-level	Items of second-level	Items of third-level	Mean	SD	CV	Approval rate %	Notes
1. Life-and-death literacy promotion in the elderly			4.909	0.294	0.060	100.000	
	1.1 Recognition of life-and-death		4.773	0.429	0.090	100.000	
		1.1.1 Related concepts of life and death	4.545	0.739	0.162	95.455	
		1.1.2 Life cycle (involving limitations of medicine)	4.545	0.596	0.131	95.455	
		1.1.3 Aging process	4.773	0.429	0.090	100.000	
	1.2 Terminal stage of the elderly		4.682	0.568	0.121	95.455	
		1.2.1 Physical symptoms of terminal stage	4.545	0.739	0.162	95.455	
		1.2.2 Psychological changes in the face of death	4.727	0.456	0.096	100.000	
2. Life-and-death concept establishment of the elderly			4.955	0.213	0.043	100.000	
	2.1 Life-and-death concepts in traditional Chinese culture		4.409	0.590	0.134	95.455	
		2.1.1 Life-and-death concepts in Confucianism	4.409	0.590	0.134	95.455	
		2.1.2 Life-and-death concepts in Taoism	4.227	0.685	0.162	86.364	
		2.1.3 Life-and-death concepts in Buddhism	4.318	0.716	0.166	86.364	
	2.2 Concept of good death		4.864	0.351	0.072	100.000	
		2.2.1 The elimination of negative emotions of death	4.818	0.501	0.104	95.455	Delete (duplicate with 1.2.2)
		2.2.2 Quality of life and quality of death	4.909	0.294	0.060	100.000	
		2.2.3 Good death in eastern and western cultures	4.409	0.734	0.167	95.455	
		2.2.4 Criteria for good death—WHO	4.409	0.796	0.181	90.909	
3. Life-and-death planning of the elderly			4.955	0.213	0.043	100.000	
	3.1 Advance directives and Wills		4.682	0.477	0.102	100.000	
		3.1.1 Concepts and Differences	4.500	0.598	0.133	95.455	Modify
		3.1.2 The utility conditions of the will	4.591	0.590	0.129	95.455	Modify
		3.1.3 Identify advance care planning and agents	4.773	0.429	0.090	100.000	
	3.2 Palliative-and-hospice care		4.955	0.213	0.043	100.000	
		3.2.1 Concept and Significance	4.636	0.492	0.106	100.000	
		3.2.2 Service objects and contents	4.864	0.351	0.072	100.000	
		3.2.3 Organizations and costs	4.636	0.581	0.125	95.455	
	3.3 Euthanasia		3.773	1.232	0.326	68.182	Delete
		3.3.1 Concepts	3.682	1.211	0.329	63.636	Delete
		3.3.2 Status both at home and abroad	3.591	1.141	0.318	63.636	Delete
	3.4 Funeral culture and bereavement		4.545	0.596	0.131	95.455	
		3.4.1 Funeral Ceremony	4.182	0.958	0.229	86.364	Delete (duplicate with 3.4.2)
		3.4.2 Chinese funeral customs	4.364	0.658	0.151	90.909	Modify
		3.4.3 Grief counseling for the bereaved	4.727	0.550	0.116	95.455	
4. Life-and-death thoughts of the elderly			4.591	0.908	0.198	95.455	
	4.1 Embrace the end of life		4.955	0.213	0.043	100.000	
		4.1.1 Life review and life values	4.955	0.213	0.043	100.000	
		4.1.2 The planning of elderly life	4.682	0.568	0.121	95.455	
	4.2 No regrets in late years		4.864	0.351	0.072	100.000	
		4.2.1 Discussion on happiness philosophy of the elderly	4.727	0.631	0.133	90.909	
		4.2.2 Four ways of emotion expressions (acknowledgment\apology\love\farewell)	4.909	0.294	0.060	100.000	

*Note*: *SD* Standard Deviation, *CV* Coefficient of variation

After the modification and improvement of the first two rounds of questionnaires and discussion of the research team, the third-round administration was conducted. Experts agreed on the content, and the scores reached consensus standards. And four experts made some additional suggestions on the details under the three-level items. For example, in “Planning before Death or Dying”, it is suggested to emphasize enlightening the spiritual needs of the elderly, not only in terms of religion, but also the respect of others, forgiveness, friendship, aesthetics, etc. An expert suggested that palliative-and-hospice care in homes should also be introduced in "Organizations and costs" of "Palliative-and-hospice care". Through the feedback of the third round of expert inquiries, the details of the content of life and death education for the elderly have been enriched and improved.

In the second and third rounds of questionnaires, point-to-point answers, modifications, or explanations to the previous round of expert opinions were presented anonymously. Through multiple rounds of Delphi, an expert consensus on life-and-death education content system for the elderly was finally reached. The content system included 4 first-level items, 9 second-level items, and 23 third-level items as shown in Table 5.

**Table 5:** Life-and-death education contents after expert consultation. (Third-round)

Items of first-level	Items of second-level	Items of third-level	Mean	SD	CV	Approval rate%
1. Life-and-death literacy promotion in the elderly			4.955	0.213	0.043	100.000
	1.1 Recognition of life-and-death		4.773	0.429	0.090	100.000
		1.1.1 Related concepts of life and death	4.727	0.550	0.116	95.455
		1.1.2 Life cycle (involving limitations of medicine)	4.727	0.456	0.096	100.000
		1.1.3 Aging process	4.909	0.294	0.060	100.000
	1.2 Terminal stage of the elderly		4.773	0.528	0.111	95.455
		1.2.1 Physical symptoms of terminal stage	4.773	0.528	0.111	95.455
		1.2.2 Psychological changes in the face of death	4.864	0.351	0.072	100.000
2. Life-and-death concept establishment of the elderly			4.955	0.213	0.043	100.000
	2.1 Life-and-death concepts in traditional Chinese culture		4.682	0.477	0.102	100.000
		2.1.1 Life-and-death concepts in Confucianism	4.727	0.456	0.096	100.000
		2.1.2 Life-and-death concepts in Taoism	4.682	0.477	0.102	100.000
		2.1.3 Life-and-death concepts in Buddhism	4.682	0.477	0.102	100.000
	2.2 Concepts of good death		5.000	0.000	0.000	100.000
		2.2.1 Quality of life and quality of death	5.000	0.000	0.000	100.000
		2.2.2 The connotation and extension of good death	5.000	0.000	0.000	100.000
		2.2.3 Good death in eastern and western cultures	4.727	0.456	0.096	100.000
3. Life-and-death planning of the elderly			4.955	0.213	0.043	100.000
	3.1 Planning before death or dying		5.000	0.000	0.000	100.000
		3.1.1 The concept and utility conditions of will	4.818	0.395	0.082	100.000
		3.1.2 The concept and content of advance directives	4.955	0.213	0.043	100.000
		3.1.3 Advance care planning	4.955	0.213	0.043	100.000
	3.2 Palliative-and-hospice care		4.955	0.213	0.043	100.000
		3.2.1 Concept and Significance	4.909	0.294	0.060	100.000
		3.2.2 Service objects and contents	4.909	0.294	0.060	100.000
		3.2.3 Organizations and costs	4.818	0.395	0.082	100.000
	3.3 Funeral culture and bereavement		4.773	0.429	0.090	100.000
		3.3.1 Chinese funeral customs	4.682	0.477	0.102	100.000
		3.3.2 Physical and mental counseling for the elderly after bereavement	4.909	0.294	0.060	100.000
4. Life-and-death thoughts of the elderly			4.909	0.294	0.060	100.000
	4.1 Clarification of life value		5.000	0.000	0.000	100.000
		4.1.1 Life review and meaning	5.000	0.000	0.000	100.000
		4.1.2 Achievement and evaluation of life goals	4.818	0.501	0.104	95.455
	4.2 No regrets in late years		4.909	0.294	0.060	100.000
		4.2.1 Discussion on happiness philosophy of the elderly	4.818	0.501	0.104	95.455
		4.2.2 Four ways of emotion expressions (acknowledgment\apology\love\farewell)	4.955	0.213	0.043	100.000

*Note*: *SD* Standard Deviation, *CV* Coefficient of variation

## Discussion

Education undertakes the functions of cultural selection, transmission, transformation, criticism, innovation, and remodeling [53]. Life-and-death education can not only convey knowledge of life and death, but also alleviate the negative emotions towards death, help people prepare for death, and improve the quality of death [18]. Based on the social background of aging, this study designed life-and-death education contents for the elderly to provide a buffer in the process of demise as well as references for other countries.

According to Terror Management Theory, life-and-death education plays a role as a mortality reminder for the elderly [54]. "Life-and-death literacy promotion in the elderly" gradually transitions the knowledge from life, life cycle and aging that are easily accepted by the elderly to end of life and death. This part aims to reduce the avoidance of death for the elderly through realistic knowledge explanations, and help the elderly face up to the problems of life and death. Proximal defenses may be activated when the elderly receive life-and-death knowledge, that is, the elderly begin to think about death consciously. And the elderly may take effective health behaviors in order to reduce perceived vulnerability and avoid death threats [55]. “Life-and-death planning of the elderly” provides the elderly with more options for end-of-life care beyond rescues. The purpose of this part is to put autonomy back into the elderly themselves and to let older adults know that they can plan for their own death. When thoughts of death are active but not conscious, distal defenses are activated. The elderly would defend against death by seeking cultural identity, meaning in life [56]. “Life-and-death concept establishment of the elderly” helps the elderly deepen their understanding of life and death from the perspective of history and culture, and initially form their own views on life and death. Through the introduction of life quality and death quality, the elderly are guided to attach importance to their own death and express their pursuit of good death. "Life-and-death thoughts of the elderly" enables the elderly to consciously explore their own value and meaning according to their life experiences, religious beliefs or cultural background. This part also discusses the source of happiness for the elderly, with the purpose of enriching the life of the elderly and improving their own wellbeing. The contents of life-and-death education for the elderly constructed in this study include 4 first-level items from the aspects of knowledge, culture and concepts, behavior planning and meaning of life.

From the experts' preference, we found that in the first round of Delphi, the experts scored the highest in “Introduction to palliative-and-hospice care”, and the two items with the approval rate reaching 100% were “Introduction to palliative-and-hospice care” and “Life-and-death concepts in Chinese traditional culture”. This shows that “palliative-and-hospice care” is the top priority that experts believe needs to be promoted to the elderly. It may be because palliative-and-hospice care can not only reduce the burden of elderly care, reduce the economic loss of the elderly, but also improve the quality of life-and-death of the elderly [57]. However, it cannot be ignored that the acceptance of palliative-and-hospice care is a medical care decision based on the cognition of life-and-death of the elderly [58]. According to behavioral decision theory, psychology is an important factor affecting people's decision-making [59]. And the influence of life-and-death culture on the psychological phenomenon and psychological process is long-term and difficult to change [60]. Therefore, we designed the concept of life-and-death from the perspective of traditional Chinese culture by analyzing the origin of the taboo of life-and-death, the neglected culture of life-and-death, the culture of life-and-death in the new era, and the development of the concept of life-and-death, this prompts the elderly to think about the concept of life and death invisibility. And “Life-and-death concepts in Chinese traditional culture” is also one of the options approved by the experts in their preferences.

Culture affects much on the formation of individual life and death attitudes and concepts, and even on the guidance of individual behavior [61]. Therefore, life-and-death education must take the local social and cultural background and the characteristics of the subjects into consideration. With reference to the contents of life-and-death education in Taiwan, China, which is well-developed [22], this study design combined the basis of traditional Chinese culture and the social reality in mainland China and generated the system of "Life-and-death concepts in traditional Chinese culture", "Good death in eastern and western cultures", "Funeral culture and bereavement", and "Four ways of emotion expressions (acknowledgment \ apology \ love \ farewell)". It contributed to the elderly to accept and recognize the educational content, and then renew their cognition and concepts. While inheriting the traditional culture, it is also reflecting on the renewal. Under additional cultural backgrounds or in different countries, this part of the content can be referred as per the actual conditions or replaced according to local conditions, thereby minimizing the restructuring work of life-and-death education for the elderly.

Compared with the life-and-death education in the past [62], the content of life-and-death education for the elderly constructed in this study is targeted at the needs of the elderly and their own characteristics. Based on the previous interview and survey needs of the elderly [43], this research integrated professional evaluation of geriatric nursing and geriatric psychologists, and generated the final content system of life-and-death education for the elderly with clear main body and refined contents. Moreover, the contents of "Aging process", "Terminal stage of the elderly", "Physical and mental counseling for the elderly after bereavement" and "Discussion on happiness philosophy of the elderly" were supplemented, which was more object-specific, systematic, and scientific, making up for the content deficiency of previous researches.

As the physical, psychological, and social characteristics of different groups are different, the contents of life-and-death education for the elderly constructed in this study were different from the related education of the rest of groups. Life-and-death education for medical and health workers focuses on palliative-and-hospice care skills [63]. The content of life-and-death education for cancer patients focuses on the acceptance of death by patients and their families and to an optimal choice of palliative-and-hospice care [24]. The contents of life-and-death education for the elderly constructed in the present research focused highly on the establishment of the death concept of the elderly and the clarification of the meaning of life, so as to help the elderly enrich their lifetime at an old age thereby improving the quality of life and death.

Under the social background of aging population, coupled with the urgent need to improve the death quality of the elderly in China, and palliative-and-hospice care pilot is being fully implemented [64], this research is accurately in line with the older population, and the scientific system of life-and-death education contents is practical and applicable [21]. We believe that life-and-death education is the right of the elderly. We can use this education to mobilize the initiative and enthusiasm of the elderly as a social resource, change the perception that the elderly is a burden on society, give full play to the social value of the elderly, and meet the self-realization needs of the elderly.

### Limitations

Limitations of this research include the failure in carrying out life-and-death education practice for the elderly, and a lack of research on the time arrangement and methodology in each part of life-and-death education. Although some of the life-and-death education program parts have not been explored, it is definite that life-and-death education for the elderly should be given in a direct way easy to be accepted and understood. This needs to be further explored in future research.

## Conclusions

The expert consensus on the content of life-and-death education for the elderly constructed in this study provides a basis for health professionals to initiate life-and-death dialogue, death preparation, and end-of-life planning for the elderly. It also plays a positive role in acceptance and implementation of palliative care in older populations. Furthermore, the result can also assist in the planning of hospice care services to improve quality of death of the elderly. This study could enrich life-and-death education resources for the elderly, and provide theoretical support for relevant research peers and palliative care professionals around the world.

## Data Availability

The data that support the findings of this study are available from the corresponding author upon reasonable request.

## References

[1] ONU (2019). World Population Prospects 2019.

[2] World Health Organization. Ageing and health. 2021. https://www.who.int/news-room/fact-sheets/detail/ageing-and-health. Accessed 12 Jan 2022.

[3] Ewa R, Paulina N, Agnieszka P et al. The World Health Organization (WHO) approach to healthy ageing. Maturitas. 2020;139:6–11. 10.1016/j.maturitas.2020.05.018 PMC7250103 32747042

[4] Bulletin of the Seventh National Census (No. 8). 2021. http://www.stats.gov.cn/tjsj/tjgb/rkpcgb/qgrkpcgb/202106/t20210628_1818827.html. Accessed 12 Jan 2022.

[5] Weir JM, Aicken MD, Cupples ME, Steele K (2015). From hippocrates to the Francis report-Reflections on empathy. Ulster Med J.

[6] He FX, Geng X, Johnson A (2021). The experience of palliative care among older Chinese people in nursing homes: a scoping review. Int J Nurs Stud.

[7] Suurmond J, Lanting K, de Voogd X (2021). Twelve tips to teach culturally sensitive palliative care. Med Teach.

[8] Pringle J, Johnston B, Buchanan D (2015). Dignity and patient-centred care for people with palliative care needs in the acute hospital setting: a systematic review. Palliat Med.

[9] Chen Q, Flaherty JH, Guo JH (2017). Attitudes of older Chinese patients toward death and dying. J Palliat Med.

[10] Nakagi S, Tada T (2014). Relationship between identity and attitude toward death in Japanese senior citizens. J Med Invest.

[11] McAfee CA, Jordan TR, Cegelka D et al. COVID-19 brings a new urgency for advance care planning: Implications of death education. Death Stud. 2022;46(1):91–6. 10.1080/07481187.2020.1821262 32941112

[12] Sinoff G (2017). Thanatophobia (Death Anxiety) in the elderly: the problem of the child's inability to assess their own parent's death anxiety state. Front Med (Lausanne).

[13] Testoni I, Palazzo L, Ronconi L (2021). The hospice as a learning space: a death education intervention with a group of adolescents. Bmc Palliat Care.

[14] Luciana MF, Ines T (2012). The emergence of thanatology and current practice in death education. Omega- J Death Dying.

[15] Wysokinski M, Fidecki W, Jarosz M (2019). Elderly people's acceptance of death: a study of a polish cohort. Int J Environ Res Public Health.

[16] The Economist Intelligence Unit. Quality of Death Index. 2015. http://www.lienfoundation.org/sites/default/files/2015%20Quality%20of%20Death%20Report.pdf. Accessed 12 Jan 2022.

[17] Zhang H, Hu M, Zeng L, Ma M, Li L (2020). Impact of death education courses on emergency nurses' perception of effective behavioral responses in dealing with sudden death in China: a quasi-experimental study. Nurse Educ Today.

[18] Doka KJ (2015). Hannelore Wass: death education–an enduring legacy. Death Stud.

[19] Smilie KD. Death education's "period of popularity": Lessons for contemporary P-12 schools in the United States during the COVID-19 pandemic. Death Stud. 2022;46(1):65–77. 10.1080/07481187.2021.1902427 33760705

[20] Fortner BV, Neimeyer RA (1999). Death anxiety in older adults: a quantitative review. Death Stud.

[21] Zhao SX, Qiang WM, Zheng XN, Luo ZQ (2018). Development of death education training content for adult cancer patients: a mixed methods study. J Clin Nurs.

[22] Phan HP, Ngu BH, Chen SC (2021). Life, death, and spirituality: a conceptual analysis for educational research development. Heliyon.

[23] Zimmermann C, Rodin G (2004). The denial of death thesis: sociological critique and implications for palliative care. Palliat Med.

[24] Testoni I, Palazzo L, Calamara N, Rossi G, Wieser MA (2021). "Imagine You Have ALS": death education to prepare for advance treatment directives. Behav Sci (Basel).

[25] Testoni I, Iacona E, Fusina S (2018). "Before I die I want to …": An experience of death education among university students of social service and psychology. Health Psychol Open.

[26] Sahan E, Eroglu MZ, Karatas MB (2018). Death anxiety in patients with myocardial infarction or cancer. Egypt Heart J.

[27] Turner V, Flemming K (2019). Socioeconomic factors affecting access to preferred place of death: a qualitative evidence synthesis. Palliat Med.

[28] Asadpour M, Sabzevari L, Ekramifar A, Bidaki R (2016). The attitude of medical students toward death: a cross-sectional study in Rafsanjan. Indian J Palliat Care.

[29] Lemaster P, Moyer E (2020). A matter of life and death: teaching undergraduates about the end of life. Int J Aging Hum Dev.

[30] Friesen H, Harrison J, Peters M, Epp D, McPherson N (2020). Death education for children and young people in public schools. Int J Palliat Nurs.

[31] Tajiri T, Matoba S, Kuwabara M (2020). Death education for future physicians. Geriatr Gerontol Int.

[32] Kim BR, Cho OH, Yoo YS (2016). The effects of Dying Well Education Program on Korean women with breast cancer. Appl Nurs Res.

[33] Testoni I, Tronca E, Biancalani G, Ronconi L, Calapai G (2020). Beyond the Wall: Death Education at Middle School as Suicide Prevention. Int J Environ Res Public Health.

[34] Wu MM. Death education from an Hegelian perspective. Death Stud. 2022;46(1):100–110. 10.1080/07481187.2020.1716885 31989866

[35] Keeney S, Hasson F, McKenna HP (2011). The Delphi technique in nursing and health research.

[36] Gregory JS, Francis TH, Jennifer K (2007). The Delphi method for graduate research. J Inf Technol Educ Res.

[37] Felicity H, Sinead K (2011). Enhancing rigour in the Delphi technique research. Technol Forecast Soc Chang.

[38] Tim CEE, Holly PK (2005). Enhancing a Delphi study on family-focused prevention. Technol Forecast Soc Chang.

[39] Hasson F, Keeney S, McKenna H (2000). Research guidelines for the Delphi survey technique. J Adv Nurs.

[40] Kennedy HP (2004). Enhancing Delphi research: methods and results. J Adv Nurs.

[41] Reimer-Kirkham S, DaCosta A, De Boer M (2020). Inclusion and quality of life for older adults: nurses advocating for policy change. Home Healthc Now.

[42] Frazer MS, Mobley P (2017). A mixed methods analysis of quality of life among late-life patients diagnosed with chronic illnesses. Health Qual Life Outcomes.

[43] Lei L, Gan Q, Gu C, Tan J, Luo Y (2022). Life-and-death attitude and its formation process and end-of-life care expectations among the elderly under traditional Chinese culture: a qualitative study. J Transcult Nurs.

[44] Woods B, Clare L (2008). Handbook of the Clinical Psychology of Ageing.

[45] Zhu M (2017). Natural Life, Health Regimen.

[46] How Palliative Care Improves Seniors’ Quality of Life. https://dailycaring.com/palliative-care-improves-seniors-quality-of-life/ (2021, Accessed 12 Jan 2022)

[47] Palliative Senior Care. https://www.seniorliving.org/palliative-care/ (2021, Accessed 12 Jan 2022)

[48] Palliative Care for Elderly. https://www.seniorguidance.org/senior-living/palliative-care/ (Accessed 12 Jan 2022)

[49] Strom M, Lonn L, Bech B, Schroeder TV, Konge L (2017). Assessment of competence in EVAR procedures: a novel rating scale developed by the Delphi technique. Eur J Vasc Endovasc Surg.

[50] China Voluntary Registration and Management System for Human Organ Donation -- FAQ. https://register.codac.org.cn/answerquestion/commonproblem.aspx (Accessed 12 Jan 2022).

[51] Balasubramaniam M (2018). Rational suicide in elderly adults: a clinician's perspective. J Am Geriatr Soc.

[52] Wu T, Chassang G (2020). Chapter 6. French and Chinese regulatory approaches to end-of-life and euthanasia. J Int Bioethique Ethique Sci.

[53] Martinez-Heredia N, Soriano DA, Amaro AA, Gonzalez-Gijon G (2021). Health education as a means of addressing death in the elderly. Int J Environ Res Public Health.

[54] Lieberman EJ (2004). Terror management theory. Am J Psychiatry.

[55] Wolfe SE, Tubi A (2019). Terror management theory and mortality awareness: a missing link in climate response studies? Wiley Interdisciplinary Reviews. Climate Change.

[56] Pyszczynski T, Greenberg J, Solomon S (1999). A dual-process model of defense against conscious and unconscious death-related thoughts: an extension of terror management theory. Psychol Rev.

[57] Lodovico B (2019). Geriatric oncology, spirituality, and palliative care. J Pain Symptom Manag.

[58] Wendy SH (2019). Patient handout: decision making & palliative care. Oncology Times.

[59] Hogarth HJEA (1981). Behavioral decision theory: processes of judgment and choice. J Account Res.

[60] Lehman DR, Chiu CY, Schaller M (2004). Psychology and culture. Annu Rev Psychol.

[61] Ke LS, Huang X, Hu WY, O'Connor M, Lee S (2017). Experiences and perspectives of older people regarding advance care planning: a meta-synthesis of qualitative studies. Palliat Med.

[62] Dickinson GE, Mermann AC (1996). Death education in U.S. medical schools, 1975-1995. Acad Med.

[63] Macdonald ME, Singh HK, Bulgarelli AF (2020). Death, dying, and bereavement in undergraduate dental education: a narrative review. J Dent Educ.

[64] Yan Y, Zhang H, Gao W (2020). Current awareness of palliative care in China. Lancet Glob Health.

